# How to build your dragon: scaling of muscle architecture from the world’s smallest to the world’s largest monitor lizard

**DOI:** 10.1186/s12983-016-0141-5

**Published:** 2016-02-18

**Authors:** Taylor J. M. Dick, Christofer J. Clemente

**Affiliations:** Department of Biomedical Physiology and Kinesiology, Simon Fraser University, Burnaby, BC Canada; School of Science and Engineering, University of the Sunshine Coast, Brisbane, QLD Australia

**Keywords:** Locomotion, Morphology, Musculoskeletal, *Varanus*, *komodoensis*, *priscus*

## Abstract

**Background:**

The functional design of skeletal muscles is shaped by conflicting selective pressures between support and propulsion, which becomes even more important as animals get larger. If larger animals were geometrically scaled up versions of smaller animals, increases in body size would cause an increase in musculoskeletal stress, a result of the greater scaling of mass in comparison to area. In large animals these stresses would come dangerously close to points of failure. By examining the architecture of 22 hindlimb muscles in 27 individuals from 9 species of varanid lizards ranging from the tiny 7.6 g *Varanus brevicauda* to the giant 40 kg *Varanus komodoensis*, we present a comprehensive dataset on the scaling of musculoskeletal architecture in monitor lizards (varanids), providing information about the phylogenetic constraints and adaptations of locomotor muscles in sprawling tetrapods.

**Results:**

Scaling results for muscle mass, pennation and physiological cross-sectional area (PCSA), all suggest that larger varanids increase the relative force-generating capacity of femur adductors, knee flexors and ankle plantarflexors, with scaling exponents greater than geometric similarity predicts. Thus varanids mitigate the size-related increases in stress by increasing muscle mass and PCSA rather than adopting a more upright posture with size as is shown in other animals. As well as the scaling effects of muscle properties with body mass, the variation in muscle architecture with changes in hindlimb posture were also prominent. Within varanids, posture varies with habitat preference. Climbing lizards display a sprawling posture while terrestrial lizards display a more upright posture. Sprawling species required larger PCSAs and muscle masses in femur retractors, knee flexors, and ankle plantarflexors in order to support the body.

**Conclusions:**

Both size and posture-related muscle changes all suggest an increased role in support over propulsion, leading to a decrease in locomotor performance which has previously been shown with increases in size. These estimates suggest the giant Pleistocene varanid lizard (*Varanus megalania priscus*) would likely not have been able to outrun early humans with which it co-habitated the Australian landmass with.

**Electronic supplementary material:**

The online version of this article (doi:10.1186/s12983-016-0141-5) contains supplementary material, which is available to authorized users.

## Background

The design of skeletal muscle is shaped by conflicting selective pressures. Muscle must perform two tasks during locomotion—support and propulsion, yet the optimal configuration to maximise one of these tasks may limit a muscles ability to perform the other. Given similar biochemical properties and sarcomere lengths, a longer muscle fibre will have both a greater working range and a higher maximal shortening velocity, increasing its ability to produce power over a range of muscle lengths to propel the body forward [1–3]. The ability of a muscle to produce force, and therefore support, is also dependent on the arrangement of fibres within a muscle. The fibres within some muscles may lie at an angle to the direction of pull (the angle of pennation), which allows a greater number of shorter fibres to be packed within a muscle, increasing the physiological cross-sectional area (PCSA), and therefore the force-generating ability of the muscle. However, this arrangement of a greater number of shorter fibres may limit a muscles ability to undergo large length changes and shorten at high velocities. Thus there is a trade-off in the design of skeletal muscle—a muscle optimized to perform one task (eg., support) may be limited in its ability to perform another (eg., propulsion) which becomes even more important as animals get larger.

The effects of size and the fundamental selective pressures associated with it have been recognized for nearly four centuries [1]. When animals move, mechanical stresses are placed on their musculoskeletal system. If larger animals were to be geometrically scaled up versions of smaller animals, increases in body size would result in an increase in the stresses (force per unit area) on muscles and bones with body mass as M^0.33^, a result of the geometric scaling of mass M^1^ and area M^0.66^ [2]. This becomes a problem for larger animals, where stresses will become dangerously closer to failure points, increasing the probability of fractures or tears [4]. In order to maintain similar magnitudes of stress, the cross-sectional area of muscles would need to scale as M^1.0^ rather than M^0.66^. McMahon [5, 6] proposed that muscles need not scale with these static stress similar proportions to maintain stresses at equivalent ratios (safety factors), but rather need to scale as elastically similar proportions. Under these conditions, lengths are expected to scale proportional to M^0.25^, diameters to M^0.375^ and cross-sectional areas to M^0.75^. Yet even these lower scaling exponents suggests that the architecture of skeletal muscle may shift toward a mechanical role in support at the cost of propulsion as size increases.

Several studies have attempted to understand the extent to which animals can, and do, change the structure of muscles with body size. Alexander [7] examined the major leg muscles in terrestrial mammals ranging from an 8 gram shrew to a 2500 kilogram African elephant which suggested an overall scaling similar to what was predicted by McMahon’s [5, 6] static stress models. However, other animals exhibited different scaling results. Within the aquatic frog *Xenopus laevis*, the isometric force produced by the plantaris muscle, responsible for producing much of the thrust during the power-stroke, was shown to scale as M^0.90^ [8], greater than the estimates for elastic similarity. Rats display scaling exponents for PCSA greater than that expected by isometry in proximal thigh muscles, the semimembranosus, vastus intermedius, and vastus medialis [9]. Ontogenetic scaling in *Alligator mississippiensis* showed that muscle mass and architecture generally scaled isometrically [10]. However for these Alligators, some exceptions to isometric scaling were shown in extensor fascicle lengths, which suggested an increase in the working range of these muscles, likely related to the greater postural variability with ontogeny [10]. Variation among taxonomic levels and life history traits, such as aquatic versus terrestrial locomotion, make it difficult to synthesize general scaling models from these comparative results.

Adding to this complexity, is the observation that some animals change posture with body size. To counter size-related increases in stress, mammals modify their biomechanical posture by becoming more upright [4, 11, 12]. This has the effect of aligning the limbs closer to the direction of the ground reaction force vector, reducing the net joint moment required to counteract external moments, consequently minimizing musculoskeletal stress during locomotion [11]. There is strong evidence to support this among phylogenetically diverse groups of mammals [11] and birds [13], and biomechanical modelling within these groups has shown elegantly how these changes can effectively counteract size-related increases in stress. However, this makes it difficult to determine if muscle architecture is responding to variation in animal size or to changes in muscle function resulting from their postural shift.

Following this idea, many studies report differences in muscle architecture in relation to function, suggesting that skeletal muscle architecture is linked to the functional requirements of movement within an animal. For example, knee flexors which produce large displacements are commonly characterized by long fascicles and small PCSAs, whereas antigravity muscles such as the soleus, generate higher forces due to their greater pennation angles and PCSAs [14–16]. This suggests that differences in muscle architecture are related to variations in body size as well as functional requirements of locomotion, which can be difficult to differentiate.

**Fig. 1:**
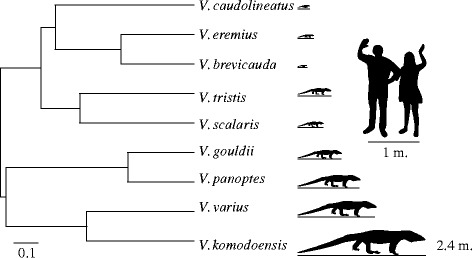
Phylogeny of varanids used in the current study, based on Thompson et al. [55], with the scale bar indicating substitutions per site. Average body lengths for each species is indicated as the length of the line under each silhouette. Approximate body size of a human is added for comparison to scale

Given the complexities described above, the most ideal group to study the effects of size and posture on the functional architecture of skeletal muscle would be a closely related group, with a known phylogeny (to reduce phylogenetic effects), which show great variation in size, and where size and posture are not correlated. Monitor (*Varanus*) lizards are the ideal paradigm to study these effects. Within a single genus, they vary in body size by nearly 5-orders of magnitude. This genus includes the world’s smallest monitor lizard—*Varanus brevicauda*, with adult body masses <10 g, and the world’s largest lizard—*Varanus komodoensis* which can weigh over 100 kg (Fig. 1). Further, postural kinematics have been described for many species in this group, and rather than being related to size as is the case for mammals [4], posture appears to be more closely related to climbing habitat [17]. Terrestrial varanids display increased femur adduction, femur rotation, and ankle range of motion in comparison to arboreal species [18]. This was explained since arboreal animals must overcome not only inertial and environmental forces, but also un-stabilizing gravitational forces when climbing inclined surfaces, and thus are more likely to adopt a crouched posture to keep their centre of mass close to their climbing substrate [19, 20].

The goal of this study is to disentangle and describe the musculoskeletal variation associated with changes in body size and those related to changes in posture. Applying these concepts specifically to varanid lizards, we predict that for larger lizards, the size-related increases in stress will be counteracted by increases in relative muscle size, force-generating ability (PCSA, pennation angle), or moment arm. Further, postural variation may also require modifications in muscle architecture. We predict arboreal varanids, when compared to terrestrial species, will require increases in relative PCSA, muscle mass, or pennation angle to counter their habitat-related crouched posture. In this study, we quantify the architectural properties of 22 upper and lower hindlimb muscles in 27 individuals from 9 species of arboreal and terrestrial varanid lizards. We used reduced major regression analysis (RMA) to determine the relationship of muscle architecture properties with body mass combined with previously published kinematic data [17, 18] to determine the correlations between posture and muscle properties. These results are then used to infer the running capability of the giant extinct monitor *Varanus* (*megalania*) *prisca*.

## Results

### Body morphology scaling

Before we determined the influence of size on muscle architecture and function, it was important to understand how the limb segments themselves responded to changes in size (Additional file 1: Table S1). We found only partial evidence for allometric scaling of body lengths in response to size. Snout-vent length (SVL) scaled less than expected 0.29 (0.27–0.32; Phylo.RMA), mostly as a result of the lower scaling of the thorax-abdomen length rather than the head-neck lengths. Hindlimb segments lengths (thigh, shank, and foot), tail length and pelvic width all scaled as expected from isometry, with an exception being pelvic height, which scaled larger than expected from isometry 0.40 (0.35–0.45).

### Average muscle properties

**Fig. 2:**
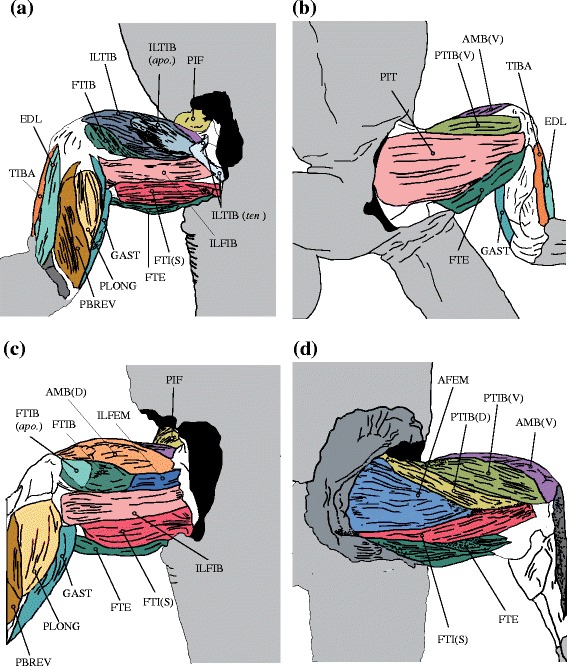
Varanid hindlimb muscle architecture. **a** Dorsal superficial, **b** Ventral superficial, **c** Dorsal deep (ILTIB removed), **d** Ventral deep (PIT removed). Apo. – Aponeurosis; D-dorsal; S-superficial; Ten. – tendon; V-ventral

Twenty-two hindlimb muscles were dissected from 27 individuals in 9 species of varanid lizards ranging from 7.6 to 40000 g (Fig. 2; Additional file 2: Table S2). Muscle origins and insertions were consistent with previous descriptions available for lizards [21–24] (Table 1) with two exceptions where our description may differ from existing literature: first, it was not possible to separate the multiple heads of the PIF so they were considered together as one muscle, and second, the PTIB was composed of 2 heads, one dorsal and one ventral, which join each other at one third from their origin, and share a common insertion, thus as above we considered these as one muscle (Fig. 2d). During the dissections, we observed a consistent pattern of aerobic and anaerobic (red and white) muscle fibre-type bands within a single muscle belly for the ILFIB, PTIB, and FTI (S) (Additional file 3: Figure S1). However, the functional reason for this regional arrangement of slow and fast muscle fibre types within an individual muscle remains unknown.

**Table 1:** Origin, insertion and function of major locomotor muscles of the varanid hindlimb based on Snyder, [21], Gans et al., [22], Reilly, [23] and Anzai et al., [24]

Muscle	Abbreviation	Function	Active during stance or swing	Origin	Insertion
Adductor femoralis	AFEM	femur adduction, femur protraction	Both^a^	puboischiadic ligament	ventral aspect of femoral shaft
Ambiens (dorsal head)	AMB (D)	knee extension	Stance	ventral rim of acetabulum	intertrochanteric notch of femur
Ambiens (ventral head)	AMB (V)	knee extension	Stance	pubis (anterior to the acetabulum)	intertrochanteric notch of femur
Caudofemoralis brevis	CFEMB	femur retraction, femur long axis rotation	Stance	posterior aspects of transverse processes of four most anterior postsacral vertebrae	fleshily, onto posterior ventral border of femoral trochanter
Caudofemoralis longus	CFEML	femur retraction, femur long axis rotation, knee flexion	Stance	proximal third of tail and 4th to 14th caudal vertebrae	broad tendon inserts onto femoral trochanter and 2nd tendon onto lateral menisci of knee
Extensor digitorum longus	EDL	ankle dorsiflexion	Swing	dorsal medial aspect of femoral epicondyle	dorsal lateral surface of 2nd and 3rd metatarsals
Femorotibialis	FTIB	knee extension	Both^a^	fleshily from entire length of femoral shaft	joins with tendon of iliotibialis to insert on cnemial crest of tibia
Flexor digitorum longus	FDL	ankle plantarflexion	Stance	femoral lateral epicondyle	Complex. Distally gives rise to a stout tendon which attaches to digits one to four
Flexor tibialis externus	FTE	knee flexion	Stance	ilioischiadic ligament	proximal lateral surface of tibia
Flexor tibialis internus (deep head)	FTI (D)	femur adduction, knee flexion	Stance^a^	posterior ventral margin of the ischium	tendon arises halfway down belly of FTI to insert onto proximal lateral surface of tibia
Flexor tibialis internus (superficial head)	FTI (S)	femur adduction, knee flexion	Both^a^	perineal region and anterior aspect of ilioischiadic ligament	inserts via 2 tendons onto proximal end of medial shaft of tibia and proximal lateral surface of tibia
Gastrocnemius	GAST	ankle plantarflexion	Stance^a^	Ventral surface of the proximal end of the tibia, and the distal end of the ventral crest of the tibia	broad aponeurosis which inserts on the proximolateral margins of the first three phalanges
Iliofemoralis	ILFEM	femur abduction	Swing	anterior aspect of blade of ilium (shares an intramuscular septum with puboischiotibialis)	belly wraps around posterior border of femur to insert into proximal aspect of posterior border of femur
Iliofibularis	ILFIB	knee flexion	Swing	fleshily from posterior ventral margin of ilium	broad flat tendon attaches onto proximal shaft of fibula (passes between peroneus longus and gastrocnemius)
Iliotibialis	ILTIB	knee extension	Stance	2 tendons with broad aponeurosis on lateral surface of ilium	inserts with femorotibialis onto cnemial crest of tibia (note the patella is imbedded in this tendon)
Peroneus brevis	PBREV	ankle dorsiflexion	Swing	broad origin on most dorsal aspects (medial and lateral) of fibula	broad tendon inserts onto the outer process of 5th metatarsal head
Peroneus longus	PLONG	ankle plantarflexion	Both^a^	short tendon from lateral femoral epicondyle	proximal end of the lateral plantar tubercle of 5th metatarsal
Puboischiofemoralis	PIF	femur protraction	Swing	anterior rim of pubic plate, pubic symphysis, ischiadic symphysis and posterior end of thyroid fenestra	femoral trochanter and posterior dorsal margin of femur
Puboischiotibialis	PIT	knee flexion	Both^a^	anterior aponeurosis and from ischiopubic ligament posteriorly	anterior medial aspect of proximal end of tibia
Pubotibialis	PTIB	femur adduction, knee flexion	Both^a^	pubic tubercle	proximal lateral aspect of tibia
Tibialis anterior	TIBA	ankle dorsiflexion	Both^a^	anterior medial aspect of tibial head and anterior dorsal surfaces of tibial shaft	ventral medial aspect of proximal end of 1st metatarsal and base of first metatarsal and metatarsophalangeal joint distally

^a^from Reilly, 1995 electromyography study [23]

Possibly the most extensive study on muscle anatomy and architectural properties in a sprawling vertebrate is that of Allen et al. [10], and we attempt to follow a similar format to facilitate comparisons among taxa. In order to make comparisons between muscles of different sizes, muscle mass, fascicle length, tendon lengths, moment arms, and PCSA data were normalized assuming geometric similarity, that is muscle mass was scaled to body mass M^1.0^ (kg), fascicle length, tendon lengths, and moments arms to M^0.33^ (kg), and PCSA to M^0.66^ (kg). Species mean normalized data for 22 muscles and 9 species are displayed in Additional file 4: Table S3.

Similar to previous studies [7, 9, 10, 25–31] we observed a proximal to distal gradient in mean muscle architectural properties across all species. The heaviest muscles of the upper limb were the CFEML (1.458 ± 0.65 % of M^1.0^, mean ± SD), PIF (0.416 ± 0.08 %), and PIT (0.336 ± 0.13 %), whereas the heaviest muscles of the lower limb were the GAST (0.285 ± 0.16 %) and the PLONG (0.107 ± 0.02 %) (Additional file 4: Table S3).

There was a slight proximal to distal reduction in fascicle length. However, the largest muscles did not always have the longest fascicles, nor did the smallest muscles have the shortest fascicles. Average fascicle lengths were slightly longer in the upper limb (mean 3.88 ± 1.17 % of M^0.33^, *n* = 16) as compared to the lower limb (mean 3.17 ± 0.72 %, *n* = 6). The muscles with the longest relative fascicle lengths were all located within the upper limb: the PTIB (5.01 ± 0.29 %), PIT (5.99 ± 0.54 %), FTI (S) (4.85 ± 0.68 %), and the CFEML (4.68 ± 0.98 %) (Additional file 4: Table S3).

Six of the twenty-two muscles analysed were pennate (pennation > 3°). Most lower limb muscles were more pennate than upper limb muscles with two exceptions, the CFEML (22 ± 1.3°) and the FTIB (19 ± 3.9°). 4 of the 6 lower limb muscles were organized in a pennate arrangement: the GAST (23 ± 2.2°), PBREV (19 ± 5.1°), PLONG (14 ± 1.9°), and the TIBA (12 ± 4.9°).

Average PCSAs were almost twice as large in the upper limb (mean 0.0084 ± 0.01 % of M^0.66^) compared to the lower limb (mean 0.0044 ± 0.003 %). In the upper limb, PCSA was greatest in the CFEML (0.039 ± 0.011 %), PIF (0.011 ± 0.003 %), and CFEMB (0.010 ± 0.003 %), whereas in the lower limb PCSA was greatest in the GAST (0.011 ± 0.003 %).

Where present, we measured tendon lengths at both the proximal and distal regions of the muscle. 16 of the 22 muscles analysed had substantial external tendons. External tendons were more prevalent in the distal limb muscles as compared to the proximal limb. Similar to Alligators there were no strong proximal to distal patterns of tendon length [10]. The longest tendon belonged to the primary insertion of the CFEML onto the femoral trochanter (2.67 ± 1.08 % of M^0.33^) and the FDL (1.37 ± 0.23 % of M^0.33^) and GAST (1.46 ± 0.07 %) displayed substantial distal tendons. The FTE (1.47 ± 0.07 %) and ILTIB (1.36 ± 0.09 %) displayed substantial proximal tendons. The ILTIB originates from 2 observable tendons which arise from the lateral surface of the ilium. ILTIB tendon length was computed as the average of these anterior and posterior tendons.

### Scaling regression analysis

**Fig. 3:**
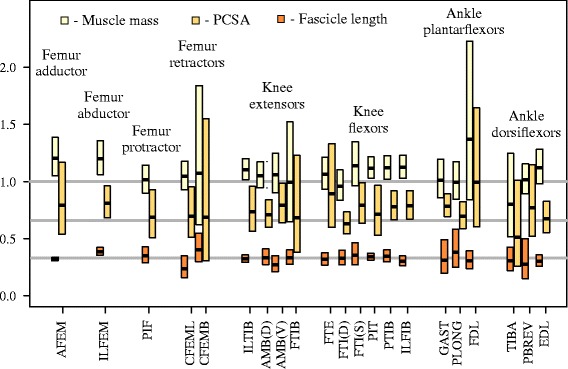
Scaling exponents for muscle properties versus body mass. The boxes represent the slopes and 95 % confidence intervals of the species mean RMA lines for log transformed muscle properties: muscle mass, PCSA, and fascicle length. Horizontal lines show predictions based on geometric scaling at M^0.33^ (length), M^0.66^ (area), M^1.0^ (mass)

The slopes and 95 % confidence intervals of the RMA lines for log transformed muscle properties versus body mass are shown in Fig. 3 and Additional file 5: Table S4. Scaling was determined to be statistically different from the expected exponent if the expected exponent fell outside these confidence intervals.

#### Muscle mass

The scaling of muscle mass with body mass was found to be highly correlated with *R*^*2*^ values above 0.9 for all muscles, even when phylogenetically informed statistics were used. Of the 22 muscles included in our analysis, 4 muscles showed significantly greater scaling of muscle mass than expected from geometry using phylogenetically informed statistics. Muscle mass scaled with exponents >1 in the AFEM (slope: 1.049–1.385), the ILFEM (slope: 1.040–1.379) and 2 of the knee flexors, the ILFIB (slope: 1.001–1.269) and the PIT (slope: 1.019–1.244). A further 4 muscles showed significantly higher scaling at the individual level, although the CI’s increased when phylogenetically informed species means were used. These included the EDL (slope individuals: 1.006–1.156; slope species: 0.944–1.363), FDL (slope individuals: 1.082–1.88; slope species: 0.84–2.229), ILTIB (slope individuals: 1.028–1.140; slope species: 0.984–1.235) and the PTIB (slope individuals: 1.019–1.288; slope species: 0.993–1.233) (Fig. 3).

#### Fascicle length

Fascicle length scaled differently than geometric expectations in only one of the 22 muscles; the ILFEM showed greater than expected scaling of fascicle length (slope: 0.351–0.420) indicating greater range of motion with increased body mass. Two muscles showed lower than expected scaling of fascicle length though not with universal agreement. The AMB (V) showed significantly lower scaling at the species level (slope: 0.208–0.348), though this was not supported for individuals (slope: 0.249–0.350), while the EDL showed significantly lower scaling at the individual level (slope: 0.261–0.322) but not at the species level (slope: 0.156–0.357) (Fig. 3).

#### Pennation angle

We expected pennation angle to scale with M^0^ however 4 muscles scaled with positive allometry. The ankle plantarflexors GAST and PLONG scaled as 0.024–0.104 and 0.038–0.140, respectively, the femur retractor CFEML scaled as 0.014–0.075 while the knee extensor FTIB scaled as 0.039–0.278, though this latter case showed large CI’s.

#### PCSA

Three muscles showed greater than the expected geometric scaling of M^0.66^ for PCSA, but did not differ from the predicted elastic similarity scaling exponent of M^0.75^. The femur abductor ILFEM scaled as 0.684–0.967, while the knee flexor, ILFIB scaled as 0.665–0.968, and the ankle plantarflexor GAST scaled as 0.702–0.939. Two other knee flexors also showed weak evidence of greater than geometric scaling. The FTI (S) scaled significantly greater than expected for individuals (slope: 0.693–0.953), but this was not supported among species (slope: 0.635–0.996). Similarly the PTIB scaled significantly higher than 0.66 among individuals (slope: 0.714–0.984), but not among species (slope: 0.639–0.928) (Fig. 3).

#### Moment arms

Moment arms did not convincingly scale different to the expectations of geometric similarity for any muscles measured. Distal moment arms were measured for 17 muscles, with the highest exponents observed for the AMB (D) (slope: 0.320–0.619), the FTE (slope: 0.324–0.594), FTI (D) (slope: 0.319–0.496) and the ILFIB slope: (0.324–0.514). Proximal moment arms were measured for 6 muscles. Of these only the EDL scaled differently than geometric expectations, though this was only significant at the individual level (slope: 0.341–0.493), and was not supported when using phylogenetically informed slopes with species means (slope: 0.316–0.432).

### Posture

To determine the influence of posture on muscle properties we used residual size-corrected muscle characteristics in relation to kinematic data (Table 2) available for varanids from previously published values in Clemente et al. [17, 18, 32].

**Table 2:** Supported correlations of muscle properties with posture variables. The sign indicates whether the relationship between kinematic and muscle properties was positive or negative

Muscle	Muscle mass	Fascicle length	PCSA	Pennation	Distal moment arm	Proximal moment arm
*Femur muscles*						
AFEM						
ILFEM		+FADD				
PIF		+HH				
CFEML		+HH, −FR	+FRo, −FR, −KA			
CFEMB						
*Knee extensors*						
ILTIB	+HH, +FA, −KA				+KA*	
AMD (D)						
AMD (V)						
FTIB	-KA	+FADD		+KA		
*Knee flexors*						
FTE						
FTI(D)	-FR, −KA		-HH, −AA		+AA	
FTI(S)	-FR, −KA, +FRo	-FR	-KA		+AA	
PIT	-FR, −KA					
PTIB	-FRo		-HH, −AA		-KA*	
ILFIB	-FR, −FRo					
*Ankle plantarflexors*						
GAST			-KA, −FR, +FADD		+AA	+AA*
PLONG	+FADD	+FRo, +FADD, −FR, −KA*, +FDep	-KA, +FADD	+FR	+FADD	+FDep
FDL						
*Ankle dorsiflexors*						
TIBA						
PBREV						
EDL	-KA, −FR	+FADD, −KA	-KA, +FADD*			

*AA* Ankle Angle, *FADD* Femur Adduction, *FDep* Femur Depression, *FR* Femur Retraction, *FRo* Femur Rotation, *KA* Knee Angle, *HH* hip height at midstance, *KA* knee angle at midstance. *P = 0.05-0.09

#### Muscle mass

None of the muscles which insert onto the femur (referred to below as femur muscles) showed a consistent relationship between muscle mass and kinematic variables. Of the knee extensors, the ILTIB showed the strongest response to changes in kinematics. There was a positive relationship between muscle mass and femur adduction meaning larger muscles were associated with a more upright stance (r = 0.78, *P* = 0.020). This was also supported by a positive association with size-corrected hip height (r = 0.86, *P* = 0.012). A weaker, and negative relationship was suggested for this muscle between muscle mass and knee angle at midstance (r = −0.73, *P* = 0.061). Of the other knee extensors, the only noteworthy association was for the FTIB which similarly had a weak association with knee angle at midstance (r = −0.88, *P* = 0.046).

Among the knee flexors, there was a strong negative association between muscle mass and femur retraction at midstance. The PIT, ILFIB, and FTI (S) all showed significant associations (r = −0.92, *P* = 0.003; r = −0.80, *P* = 0.016; r = −0.97, *P* = 0.030 respectively) with the FTI (D) showing the weakest association (r = −0.94, *P* = 0.051). Three of these muscles FTI (D), FTI (S) and the PIT also showed a negative association with the knee angle at midstance (r = −0.96, *P* = 0.036; r = −0.99, *P* = 0.008; r = −0.85, *P* = 0.015 respectively). There was also evidence for an association with these muscles and femur rotation; the FTI (S) showed a positive association with femur rotation at midstance (r = 0.95, *P* = 0.044), whereas both the ILFIB and the PTIB showed a negative relationship with the change in femur rotation during the stance phase (r = −0.84, *P* = 0.015; r = −0.96, 0.035 respectively).

The ankle muscles show less association with kinematics. The ankle plantarflexor PLONG had a positive correlation with femur adduction (r = 0.99, *P* = 0.028) whereas the ankle dorsiflexor EDL showed a negative relationship between muscle mass with knee angle and femur retraction at midstance (r = −0.87, *P* = 0.010; r = −0.86, *P* = 0.012).

#### Fascicle length

Among the femur retractor muscles the PIF showed relatively longer fascicles which are linked with relatively higher hip heights at midstance (r = 0.93, *P* = 0.022). The CFEML showed a negative association of fascicle length with the change in femur retraction (r = −0.97, *P* = 0.025). In contrast, longer fascicles of the femur abductor ILFEM were associated with greater angular changes in the abduction/adduction axis of the femur (r = 0.97, *P* = 0.026).

Among knee extensors only the FTIB showed a strong response with kinematics. Longer fascicles were associated with greater femur adduction (upright posture) for this muscle (r = 0.91, *P* = 0.032). Similarly, fascicle length of the knee flexors show little association with kinematics, with the exception of the FTI (S) which displayed evidence for a negative relationship between the change in femur retraction and fascicle length (r = −0.92, *P* = 0.03).

The ankle plantarflexor PLONG showed multiple associations with kinematic variables. Longer fascicles were associated with greater femur rotation (r = 0.95, *P* = 0.012) and greater femur adduction at midstance (r = 0.97, *P* = 0.030), but were negatively correlated to femur retraction at midstance (r = −0.92, *P* = 0.027). This could be related to the weak negative association of fascicle length with knee angle (r = −0.87, *P* = 0.054) or the weak positive association with the change in femur depression (r = 0.88, *P* = 0.045) this same muscle exhibited. The antagonist to this muscle the ankle dorsiflexor EDL also showed some association with kinematics. Fascicle lengths for this muscle were positively associated with femur adduction (r = 0.92, *P* = 0.028) similar to the PLONG, and negatively associated with the change in knee angle over the stance phase (r = −0.90, *P* = 0.035).

#### Pennation angle

Of the 6 muscles which showed pennation angles >3°, only two showed any strong association with kinematics. The knee extensor FTIB showed a significant positive association with the change in knee angle during the stance phase (r = 0.97, *P* = 0.045). The ankle plantarflexor PLONG displayed a positive relationship between the change in femur retraction and pennation among species (r = 0.90, *P* = 0.037).

#### PCSA

When considering PCSA, the largest muscle of the hindlimb, the femur retractor muscle CFEML showed several associations with kinematics. PCSA was positively associated with femur rotation at midstance (r = 0.94, *P* = 0.013) suggesting greater muscle force produced by the CFEML results in an increased clockwise rotation of the femur at midstance. Further, both femur retraction at midstance (r = −0.95, *P* = 0.011) and knee angle at midstance (r = −0.97, *P* = 0.025) were negatively associated with PCSA. This suggests that an increase in femur protraction and decrease in knee angle (i.e. crouched, anteriorly outstretched hindlimb at midstance) is associated with an increase in the force-generating capacity of the CFEML.

None of the knee extensors showed convincing associations between PCSA and kinematics, though a few of the knee flexors did. Further, in these cases a similar pattern emerges. The PTIB showed negative associations with ankle angle at midstance (r = −0.95, *P* = 0.046). Similarly there was evidence for a weak negative association between knee angle at midstance with PCSA for the FTI (S) (r = −0.95, *P* = 0.048). In each case, this suggests that a more crouched posture, as displayed through smaller joint angles, is associated with larger PCSAs.

This trend continues among the ankle plantarflexors and ankle dorsiflexors. The GAST shows a negative association between PCSA and knee angle at midstance (r = −0.89, *P* = 0.039), along with a negative association with femur retraction at midstance (r = −0.91, *P* = 0.032), though the association of this latter variable with posture remains unclear. The change in knee angle over the stance phase was negatively associated with PCSA for both the ankle plantarflexor PLONG (r = −0.95, *P* = 0.044) and the ankle dorsiflexor EDL (r = −0.89, *P* = 0.041). Perhaps most curious is the positive association between the GAST and PLONG and femur adduction (r = 0.95, *P* = 0.044; r = 0.94, *P* = 0.056) which is also weakly present in the EDL (r = 0.81, *P* = 0.095). These results may suggest a transition towards an increased functional importance for the distal muscles during the propulsion phase with a more upright posture.

#### Moment arms

Of the 17 muscles for which we were able to measure a distal moment arm, only 6 showed a significant correlation with posture. None of the femur muscles showed an association of moment arm with posture, but the knee extensor ILTIB did show a significant positive relationship with knee angle at midstance (r = 0.92, *P* = 0.028), though this result was not supported by independent contrasts (IC) (r = 0.90, *P* = 0.095). The knee flexors FTI (D) and FTI (S) both show a similar positive relationship with the change in ankle angle throughout the stance (r = 0.96, *P* = 0.039; r = 0.98, *P* = 0.012) as did the ankle plantarflexor GAST (r = 0.96, *P* = 0.041). Another knee flexor the PTIB showed a negative relationship of distal moment arm with the change in knee angle throughout stance (r = −0.93, *P* = 0.021), though support was weaker using IC (r = −0.94, *P* = 0.054). Finally the ankle plantarflexor PLONG showed a positive association with femur adduction (r = 0.98, *P* = 0.016).

Among the 5 muscles for which we recorded proximal moment arms, only two significant interactions with posture were evident, both among ankle plantarflexors. The GAST showed a positive association with ankle angle change throughout the stance (r = 0.95, *P* = 0.023) similar to results for the distal moment arm above, though these results were weaker when using IC (r = 0.93, *P* = 0.065). Another ankle plantarflexor PLONG showed a positive relationship between proximal moment arm and the change in femur depression (r = 0.97, *P* = 0.028).

#### Tendon lengths

None of the 16 muscles for which we measured substantial external tendons showed a significant relationship with body mass among the species measured. This may reflect the difficulty in accurately determining the boundary between aponeurosis and external tendon.

## Discussion

Variation in muscle architecture was found not only along the length of the hindlimb but also within individual muscles across different species which vary in both size and posture. The significance of this muscle variation at different levels of organisation is discussed in the context of the various functional demands placed on the musculoskeletal system. These patterns linking form and function of musculoskeletal design can be used to gain insight into the likely performance capabilities of extinct or inaccessible taxa.

### Distribution of muscle properties

Muscles in the upper limb generally had longer fascicles arranged at lower pennation angles whereas lower limb muscles had slightly shorter fascicles arranged at higher pennation angles. Muscles specialized to undergo large changes in length (i.e. joint range of motion) are typically characterized by long fascicles at low pennation angles, where more sarcomeres are arranged in series [15, 33]. Four of the six lower hindlimb muscles were organized in a pennate arrangement (range: 12–23°). The regionalization of pennate muscles in the distal limb is consistent with results of scaling studies in the rat and alligator [9, 10]. These characteristics are likely related to the greater functional range of motion at the hip joint, in comparison to the knee and ankle joints for sprawling animals, combined with the need to generate large amounts of muscular force at the ankle joint [18, 34].

On average PCSA was greater in the upper limb as compared to the lower limb, reflecting the large PCSAs for the femur retractors (CFEML and CFEMB) and femur protractor (PIF). However, a comparison of average PCSA between the upper and lower limb, excluding these three muscles, reveals that average PCSA is quite similar between regions (upper limb mean = 0.0049 % of M^0.66^; lower limb mean = 0.0044 %). The significance of these large muscles is consistent with other large sprawling tetrapods. In alligators, it has been suggested that the two most powerful muscles are the CFEML and the PIF [10], and in crocodiles, evidence suggests the CFEML is the ‘prime mover’ of the hindlimb [35, 36]. The CFEML consists of two insertion sites, onto both the femur and the tibia, which provide the propulsive power to retract the entire hindlimb during the stance phase, whereas the PIF functions during swing phase to protract the limb [23, 35, 37].

#### Scaling of muscle properties

Patterns of positive allometry in particular hindlimb muscles in larger varanids suggests an increase in their relative size, presumably to reduce the body mass related increases in stresses placed on bones and muscles. We report muscle properties where values scale differently from the predictions of geometric similarity, in most cases these better resemble the predicted scaling based upon elastic similarity [5, 6] (Additional file 5: Table S4).

**Fig. 4:**
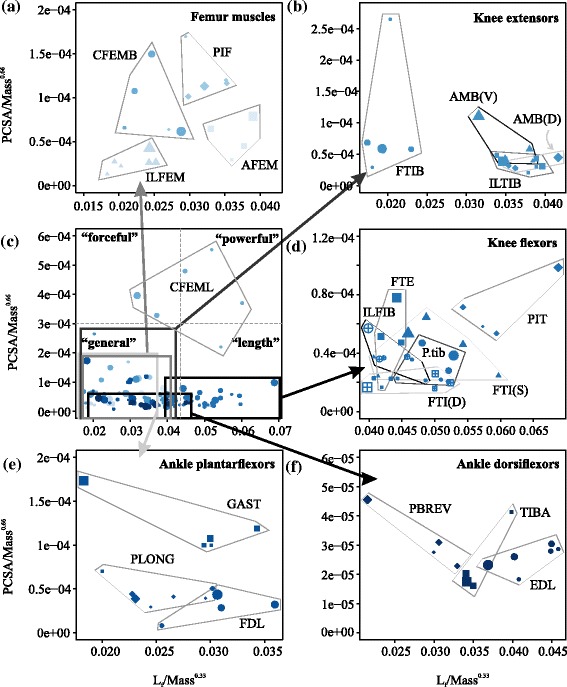
Performance space plot (fascicle length (L_f_) verses PCSA, normalised by the appropriate scaling exponents for body mass) for each hindlimb muscle. Species means are represented for each muscle where the size of the symbol indicates the mean body mass and the shape of the symbol represents different muscles for each species. A general overview for all muscles is shown in **c**, and is separated into functional groups in panels **a**-**b**, **d**-**f**

In some proximal femur muscles of larger varanids, there is likely an increase in the relative force-generating capacity of the AFEM and ILFEM— both muscles scale with positive allometry for muscle mass while the ILFEM also scales with positive allometry for PCSA. The AFEM functions in femur adduction and protraction whereas the ILFEM provides a role in femur abduction. Previous results show that hip joint angles remain unchanged across varanids, likely resulting in greater hip moments for larger species [18]. The greater scaling exponents of this agonist–antagonist muscle pair, scale in a similar manner to counteract the added torque about opposite sides of the hip joint. This can be seen by the similar size-related shifts in performance space for the AFEM and ILFEM (Fig. 4a). Similar results have been shown in mammals—proximal muscles have greater than predicted scaling exponents, suggesting a requirement for an increase in the relative force-generating capacities for certain muscles [9].

Pennation scales with positive allometry in the CFEML, one of the greatest force-generating muscles of sprawling animals [10, 35, 36]. This suggests that as varanids increase in body mass, there is a functional shift in CFEML muscle architecture towards force production over range of motion, favouring support over propulsion as shown by the directional shift in muscle performance with size within Fig. 4c. These results are in contrast to those of Allen et al., [10], who found an increase in CFEML fascicle length and decrease in PCSA with ontogeny, reflecting an ontogenetic increase in the range of motion for alligators. It is unknown whether the working range of muscles changes with ontogeny in varanids, but this disparity may be due to the habitat-related effects within aquatic versus terrestrial organisms.

Again, this pattern towards force production over range of motion is repeated among knee muscles. Muscle mass in two knee flexors, the ILFIB and PIT, scales with greater values than geometric similarity predicts. Increases in muscle mass may result from both changes in either fascicle lengths or PCSA. The PCSA of the ILFIB shows a relative increase with body size, and similarly among the other knee flexors, PCSA exponents increase by a greater magnitude than fascicle length (Fig. 3), likely driving the observed increase in mass. This provides evidence that the knee flexors show an increase in the relative force-generating capacity in larger varanids (Fig. 4d). Knee flexors are predominantly active during the swing phase of locomotion [23], thus these results may reflect the additional muscle force required to move a heavier limb.

Ankle muscles show significant scaling relationships consistent with femur and knee muscles (Fig. 3). In particular, the GAST scales with positive allometry in PCSA which may suggest that with increased body mass, this muscle increases its cross-sectional area to offset the size-predicted increase in mechanical stress (Fig. 4e). Among large varanids, the GAST may perform more than one mechanical function as it possesses relatively long fascicles, and a high PCSA as compared to all other lower limb muscles (Additional file 4: Table S3). It appears to have become specialized to produce large amounts of mechanical power (work per unit time), by both an increase in PCSA for large forces, and relatively long fascicle lengths for high velocity contractions. This suggests it provides a role during both support and propulsion. To counteract this increase in ankle plantarflexion force the antagonist muscle EDL also increases (although not significantly when using phylogenetically informed tests) in mass to values greater than geometric scaling predicts.

Pennation is not expected to scale, however the GAST and PLONG scaled with positive allometry. The pennate architecture of muscles allows more fibres to be packed into a given volume, thus increasing PCSA, and the force-generating ability of a muscle [3, 14, 15]. The GAST and PLONG are both ankle plantarflexors which are active during the stance phase to propel the lizard forward [23]. Although already designed to produce large amounts of force over a small working range (short, pennate fibres), these changes in architecture suggest that these muscles become even more specialized to deal with the size-related functional demands.

It has been suggested that the decrease in fascicle length in distal muscles of larger animals is coupled with the appearance of long elastic tendons to allow for similar joint ranges of motion [7]. Although we do not see a significant decrease in fascicle length with increases in body mass, GAST PCSA scales with positive allometry, which suggests that a greater number of short fibres are being packed into this muscle as varanids increase in size (Fig. 3). This finding also highlights the need to consider, more thoroughly, the mechanical function of the GAST tendon, and whether it plays a role in elastic energy savings with increases in body size.

Muscles that are active in swing phase seem to dominate the scaling trends seen here. Many of these muscles scaled with positive allometry with the exception of the large ankle plantarflexors, GAST and PLONG and the powerful femur retractor, CFEML. The greater force-generating capacity of swing phase muscles in larger varanids suggests that musculoskeletal adaptations with body mass are not only limited to muscles active during the stance phase of locomotion, but are also tightly linked with muscles active during the swing phase. Larger varanids may require swing phase muscles capable of generating greater forces to swing a heavier limb and overcome the added inertial effects of increased hindlimb mass [38, 39]. Alternatively, these muscles may have an additional role of support or propulsion during the stance phase to accommodate the size-related increases in mechanical stress. This would allow more muscles to be active during stance and thus operate within reasonable safety factors to avoid muscle damage [40, 41]. However, an assessment of the muscle excitation patterns during locomotion across a wide range of body sizes would be necessary to determine if, in fact, this is true. Changes in body size may be accompanied by alterations in not only the architecture, as shown here, but also the intrinsic mechanical properties of skeletal muscle. This has been shown in lacertid lizards, where there was a greater than expected increase in normalised muscle power output with body length, which is thought to result from alterations in muscle fibre density and/or changes in muscle fibre type [42].

### Variations in muscle properties with posture

While we found scaling effects of muscle properties with body mass, the variation in muscle architecture with changes in posture were even more pronounced. Among femur muscles the PCSA of the CFEML was positively associated with femur rotation at midstance. The primary tendon of the CFEML inserts onto the ventral surface of the femur causing not only femur retraction, but also long axis rotation [21, 35, 43]. Thus the increase in clockwise femur rotation, is likely a consequence of the CFEMLs need to generate larger retraction forces, resulting in greater torques about the long axis for the femur. PCSA was also negatively associated with knee angle at midstance, suggesting that a larger CFEML force-generating ability is associated with smaller knee angles. Data from Clemente et al. [18] suggests that crouched arboreal lizards have smaller knee angles in comparison to upright terrestrial species (see Fig. 3 of [18]), suggesting a shift in the musculoskeletal properties of the CFEML with posture. These results are consistent with previous findings which have compared musculoskeletal adaptations with habitat variation. Among four species of geckoes, larger femur retractor muscles were associated with the sprawling posture within arboreal species [20].

Overall, the knee flexors showed the most obvious postural-related shifts in musculoskeletal architecture. Five muscles function during swing phase to flex the knee: PTIB, PIT, ILFIB, FTI (S), and FTI (D). The negative association of muscle mass in PIT, FTI (D), and FTI (S) with knee angle at midstance suggests that these muscles increase their force-generating capacity in a more crouched posture. Similarly, the FTI (S) showed an increase in relative PCSA with smaller knee angles. Together, these results present strong evidence towards a posture-related shift in musculoskeletal architecture within knee flexors of varanid lizards. Larger knee flexors, with greater cross-sectional areas, capable of generating higher forces, allow these muscles to counteract the posture-related increase in musculoskeletal stress placed on large crouched lizards.

The ankle plantarflexors PLONG and GAS, and dorsiflexor EDL all displayed a relative increase in PCSA with smaller knee angles. The EDL also shows increased muscle mass associated with decreased knee angles at mid stance. Smaller joint angles are representative of a crouched posture, so like the knee flexors, we again see evidence for a posture-related shift in musculoskeletal architecture towards larger muscles with greater PCSAs within crouched varanids. This likely allows these large crouched lizards to operate within safe limits of musculoskeletal stress during locomotion.

Variation in fascicle length of femur muscles could also be attributed to habitat-associated variation in posture. The femur retractors PIF showed relatively longer fascicles in relation to higher hip height at midstance. Higher hip height is characteristic of a more upright posture, reflective of a terrestrial habitat [17, 18]. Evidence suggests that terrestrial and climbing varanids use different speed modulation strategies to increase running speed. Terrestrial varanids increase running speed through an increase in stride length, whereas climbing varanids increase speed via stride frequency [18], similar to results seen in geckos [20]. The shift from stride length to stride frequency speed modulation is thought to avoid instability on vertical surfaces [18]. Relatively longer fascicle lengths with upright posture may reflect this habitat-related functional change in joint range of motion for terrestrial versus climbing species.

Further, other associations between posture and fascicle length were evident. An increase in ILFEM fascicle length was linked with greater angular changes in the abduction/adduction axis of the femur. The ILFEM functions to abduct the femur during the swing phase, so an increase in relative fascicle length with range of motion is reasonable. The ankle agonist–antagonist muscle pair, the EDL and PLONG, both show an increase in relative fascicle length with increased femur adduction. Upright, terrestrial varanids display an increase in femur adduction and ankle range of motion in comparison to crouched arboreal species [18], suggesting an indirect musculoskeletal change related to this kinematic difference. The direct association between these muscles and ankle angle in our analysis does not show strong statistical support but this may be related to the difficulty in measuring ankle angles from passive marker tracking in small lizards.

## Conclusions

Here, we provide a comprehensive and quantitative dataset on the scaling of skeletal muscle architecture in climbing and non-climbing varanid lizards. This dataset includes animals across a 5-fold size range within a single genus—providing information about the phylogenetically constrained musculoskeletal adaptations that allow functional diversification across locomotor hindlimb muscles in relation to size and posture.

In comparison to an upright posture, crouched animals suffer increases in musculoskeletal stress because larger joint moments are required to counteract the external moment. Varanids do not display the same shifts towards an upright posture with size [18] as shown in other animals [11, 13]. The lack of a strong relationship between posture and body size in varanids is likely a result of an upright posture causing an increase in femur torsion, as shown among other sprawling animals [43]. Instead, it appears that crouched varanids compensate for the posture-related increases in stress by increasing muscle size and PCSA.

However, increases in the support role of muscles are likely to come at the cost of range of motion and maximum shortening speed. This conflict between support and propulsion becomes more important as animals get larger, and has the potential to decrease locomotor performance. This both explains and supports the decrease in sprint speeds relative to body size previously reported for large varanids including *V. komodoensis* [44, 45]. Both phylogenetic and physical constraints combine to produce this decrease in locomotor performance which seems likely to extend towards even larger body sizes than reported within the current study.

The largest varanid reported from fossil evidence is *Varanus* (*megalania*) *prisca* which occupied the Australasian landmass during the Pleistocene era less than 40, 000 ybp [46]. Recent evidence suggests that this species likely co-habitated this landmass with the indigenous Australians [47–49], and therefore the question of who was the hunter and the hunted between lizard and man becomes relevant. Body mass estimates for this species are reported to be up to 620 kg [46], though a more conservative estimate suggested a body mass of 330 kg [50]. However, even given this lower estimate of body size, the probable sprawling posture of this animal necessitates a decrease in performance. This decrease in performance is not only supported by our predictive scaling of muscle properties but also from previous estimates of sprint speed in varanids [44], though musculoskeletal modelling estimates are necessary to confirm this. The low estimates of performance, if indeed true, seem to support previous hypotheses that this species was more likely a scavenger than a hunter [50]. This also suggests that *Varanus* (*megalania*) *prisca* may have been particularly vulnerable to predation and hunting practices of early indigenous Australians. These low performance estimates support a growing body of evidence suggesting an increased potential of early humans towards amplifying extinctions of megafauna within the Pleistocene [47–49].

## Methods

### Specimens

For this study, we collected architectural properties of 22 upper and lower hindlimb muscles for 27 individuals from 9 species of varanids (Table 1, Additional file 2: Table S2). All specimens, with the exception of *Varanus komodoensis*, were wild caught using a variety of pit trapping and hand foraging techniques. Individuals that appeared to be sick, injured, or obviously malnourished were not included. The 3 *Varanus komodoensis* were obtained from the Queensland Museum (frozen specimens). The vast majority of our specimens were males, and no systematic differences between males and females were discovered. Therefore, sex was not differentiated in our analysis. Lizards were collected under permits WISP11435612 (QLD) and SF009075 (WA) and ethics SBS/195/12/ARC (QLD) and RA/3/100/1188 (WA).

### Muscle architecture

A standard protocol for muscle architecture was followed [10, 31, 51, 52]. Muscle belly length and external tendon lengths (m) were measured. Where present, both proximal and distal tendons were measured. Fascicle lengths (m) and pennation angles (°) were measured from a representative number of fascicles in the middle, proximal, and distal regions of the muscle belly. Mean fascicle length and pennation angle from the 3 regions were used in the statistical analysis. Individual muscles were dissected free, and muscle mass (kg) was measured. Moment arms (m) were quantified with callipers as the perpendicular distance from the joint axis of rotation to the muscles line of action. Proximal and distal moment arms were recorded for biarticular muscles. PCSA was estimated as:

1 $$ \mathrm{PCSA}=\frac{\frac{{\mathrm{v}}_{\mathrm{m}}}{{\mathrm{L}}_{\mathrm{f}}}}{ \cos \kern0.5em \uptheta} $$

Where V_m_ is the volume of muscle defined as muscle mass divided by estimated vertebrate muscle density (1.06 g cm^−3^, [53], L_f_ is fascicle length, and θ is pennation angle.

### Statistical analysis

We divided our analysis up into two parts, an analysis of the scaling of muscle properties with size, and an analysis of the variation in muscle properties with posture-related kinematic variables. We further subdivided each of these analyses into two parts. Due to logistical reasons, the number of individuals we could include in our analysis was greater than the number of species, yet individuals do not form independent points in a statistical analysis. Thus, for both tests on scaling we performed the analysis twice, first to include all individuals from all species, and the second analysis to include only species means in phylogenetically informed statistical tests. This method forms a suitable compromise between making a type II statistical error (reporting no effect where one exists), and a non-violation of the independence of data points in statistical analyses. Where results agree, we report the findings with confidence, yet where they do not, we suggest caution in the interpretation of results, which are discussed in more detail.

#### Scaling

We used RMA to determine the relationship of muscle architecture properties with body mass. RMA has the advantage of including variation in both the predictor and dependent variables in regression analysis, though some statisticians believe there are cases where this analysis may not be optimal. In our initial analysis we included all individuals from all species in RMA using the sma.R function from the SMATR package in R (R core development team 2012). Secondly, we performed a phylogenetically informed analysis on species means using the phyl.RMA.R function from the phytools package [54]. Significant differences from geometrical scaling expectations were determined using the 95 % confidence intervals from each function. For our phylogeny we used branch lengths and branching patterns based on a maximum likelihood tree from 1038 bp of the NADH-2 gene from Thompson et al. [55] (Fig. 1). The branch tips were set to unity using the chronos.R function, and the tree was pruned using the drop.tip.R function both from the Ape package [56] in R.

#### Posture

To determine the effect of posture on muscle architecture we used previously published kinematic data in Clemente et al. [17, 18, 32]. Thus, unlike the scaling analysis above, we did not have information on individual variation therefore the analysis was performed using only species means. To remove the effects of size we used the function phyl.RMA.R from the phytools package. Residual muscle properties were then correlated with kinematic variables using the cor.test.R function in R. To remove the effects of phylogenetic relatedness we also performed this analysis using phylogenetic independent contrasts (IC) calculated using the pic.R function from the ape package in R. As above where results agree we report IC results but where they differ we report both.

We tested 10 different kinematic variables related to posture. Details describing their derivation are reported elsewhere [17, 18, 32] and only a brief description of their functional significance is given here. Knee angle represents the ventral facing angle between the femur and tibia, with smaller values indicating greater flexion. The ankle angle was dorsal aspect between the foot and the tibia, with greater values indicating increased plantarflexion. Movement of the femur relative to the pelvis was described by three angles. Femur adduction described the angle of femur with a horizontal plane passing through the hip, where greater values indicate the knee is pushed further below the hip. Femur retraction was the angle between the femur and a line passing along the long axis of the pelvis (sagittal plane), such that greater values indicate greater retraction of the femur. Femur rotation is the angle between a plane containing the femur and tibia and a vertical reference plane passing through the hip and knee, where greater values indicate greater clockwise rotation. For each kinematic variable we included two values in our analysis; the angle of the joint at midstance, as well as the maximum angular excursion throughout the stance phase. Finally, we included size-corrected (from mass) hip height at midstance for each species, to give an overall estimate of posture. Given the low sample sizes, we have followed the advice of Nakagawa [57] and have not included correction for multiple comparisons. Instead we have reported correlations which have the highest r value. Individually all reported correlations have *P* values < 0.05, but the sample size is too small to adjust without making type II errors.

## Additional files

Additional file 1: Table S1. Body segment scaling exponents (*n* = 27). (PDF 271 kb) Additional file 2: Table S2. Specimen segment lengths and masses (*n* = 27). (PDF 220 kb) Additional file 3: Figure S1. Different patterns of variation in aerobic and anaerobic muscle fibres within a single muscle belly for the iliofibularis (a-b), pubotibialis (c-d) and flexor tibialis internus (superficial) (e). Although not explicitly tested, the different bands of red and white muscle fibres suggest a regional arrangement of slow and fast muscle fibres, while the functional reason for this remains unclear. (PDF 1276 kb) Additional file 4: Table S3. Average muscle properties in the hindlimb of the varanid lizards. Pennation is an angle measured in degrees. (PDF 308 kb) Additional file 5: Table S4. Results of RMA linear regression properties with body mass in the hindlimb of varanid lizards. Data presented is from a phylogenetically informed analysis on species means. (PDF 296 kb)
