# Supplementary material for: How to build your dragon: scaling of muscle architecture from the world’s smallest to the world’s largest monitor lizard
Source: Front Zool. 2016 Feb 18;13:8. doi: 10.1186/s12983-016-0141-5 (PMC4758084; doi:10.1186/s12983-016-0141-5)
Supplement: Additional file 1: Table S1. — Body segment scaling exponents (n = 27). (PDF 271 kb) [file 12983_2016_141_MOESM1_ESM.pdf]

	Head-neck Length	Thorax-abdomen Length	Snout-vent Length	Tail Length	Proximal hindlimb Length	Distal hindlimb Length	Foot Length	Pelvis Width	Pelvis Height
Slope	0.317	0.294	0.297	0.317	0.359	0.363	0.323	0.374	0.399
Lower 95% CI	0.266	0.262	0.277	0.235	0.312	0.304	0.277	0.297	0.353
Upper 95% CI	0.377	0.331	0.319	0.428	0.412	0.432	0.377	0.473	0.450
R^2	0.962	0.983	0.994	0.884	0.976	0.961	0.970	0.931	0.981
P-value	0.598	0.055	0.011	0.763	0.200	0.250	0.759	0.246	0.009
