# Supplementary material for: How to build your dragon: scaling of muscle architecture from the world’s smallest to the world’s largest monitor lizard
Source: Front Zool. 2016 Feb 18;13:8. doi: 10.1186/s12983-016-0141-5 (PMC4758084; doi:10.1186/s12983-016-0141-5)
Supplement: Additional file 2: Table S2. — Specimen segment lengths and masses (n = 27). (PDF 220 kb) [file 12983_2016_141_MOESM2_ESM.pdf]

| species | habitat | body mass (g) | head-neck length (mm) | thorax-abdomen length (mm) | SV length (mm) | tail length (mm) | thigh segment length (mm) | crus segment length (mm) | pes segment length (mm) | pelvis width (mm) | pelvis height (mm) |
|---|---|---|---|---|---|---|---|---|---|---|---|
| V. brevicauda | terrestrial | 7.6 | 25.2 | 67.8 | 93.0 | 80.0 | 8.0 | 8.8 | 11.1 | 8.8 | 6.3 |
| V. brevicauda | terrestrial | 10.0 | 20.2 | 71.8 | 92.0 | 89.1 | 7.5 | 8.4 | 11.1 | 8.6 | 5.9 |
| V. caudolineatus | arboreal | 11.1 | 32.7 | 69.3 | 102.0 | 128.9 | 10.3 | 11.6 | 13.2 | 8.5 | 6.1 |
| V. caudolineatus | arboreal | 15.0 | 40.8 | 66.2 | 107.0 | 128.1 | 11.0 | 12.8 | 15 | 8.5 | 6.5 |
| V. eremius | terrestrial | 11.8 | 37.9 | 66.1 | 104.0 | 186.0 | 10.1 | 12.5 | 18.5 | 8.7 | 7.3 |
| V. eremius | terrestrial | 20.9 | 44.0 | 83.0 | 126.0 | 216.0 | 13.7 | 18.5 | 19.5 | 9.5 | 9.4 |
| V. scalaris | arboreal | 158.1 | 72.4 | 151.2 | 223.6 | 291.0 | 24.9 | 25.4 | 37.4 | 18.1 | 17.3 |
| V. tristis | arboreal | 104.5 | 82.0 | 140.0 | 222.0 | 417.0 | 31.9 | 32.3 | 35.3 | 17.4 | 17.1 |
| V. tristis | arboreal | 104.5 | 73.0 | 127.0 | 200.0 | 406.0 | 23.8 | 22.0 | 37.2 | 19.6 | 16.3 |
| V. tristis | arboreal | 265.0 | 106.0 | 166.0 | 272.0 | 428.0 | 31.4 | 34.2 | 38.8 | 19.3 | 24.2 |
| V. gouldii | terrestrial | 429.4 | 105.0 | 203.1 | 308.1 | 542.1 | 34.6 | 45.3 | 59.4 | 30.3 | 27.1 |
| V. gouldii | terrestrial | 439.8 | 83.0 | 207.0 | 290.0 | 488.2 | 33.3 | 44.4 | 54.2 | 28.8 | 23.6 |
| V. gouldii | terrestrial | 459.3 | 156.0 | 234.0 | 390.0 | 498.0 | 33.2 | 48.9 | 45.8 | 29.2 | 25.4 |
| V. panoptes | terrestrial | 15.3 | 49.3 | 63.7 | 113.0 | 165.0 | 13.1 | 16.4 | 22.8 | 8.9 | 7.3 |
| V. panoptes | terrestrial | 661.2 | 136.0 | 256.0 | 392.0 | 602.0 | 49.6 | 59.4 | 71.8 | 36.2 | 35.2 |
| V. panoptes | terrestrial | 724.0 | 153.0 | 232.0 | 385.0 | 521.0 | 43.5 | 52.4 | 46.2 | 28.8 | 33.6 |
| V. panoptes | terrestrial | 979.3 | 151.0 | 251.0 | 402.0 | 589.0 | 65.0 | 70.2 | 67.9 | 35.3 | 38.0 |
| V. panoptes | terrestrial | 1059.9 | 139.0 | 269.0 | 408.0 | 565.0 | 62.1 | 68.1 | 63.5 | 37.8 | 38.7 |
| V. panoptes | terrestrial | 2076.8 | 161.1 | 322.3 | 483.4 | 727.1 | 59.8 | 74.0 | 83.8 | 36.2 | 49.0 |
| V. panoptes | terrestrial | 4150.0 | 219.0 | 371.0 | 590.0 | 686.0 | 72.0 | 97.4 | 82.8 | 67.7 | 64.4 |
| V. varius | arboreal | 810.7 | 82.0 | 140.0 | 222.0 | 417.0 | 31.9 | 32.3 | 35.3 | 17.4 | 17.1 |
| V. varius | arboreal | 834.4 | 154.7 | 242.0 | 396.7 | 649.0 | 54.0 | 52.9 | 77.5 | 34.8 | 37.1 |
| V. varius | arboreal | 4025.2 | 214.0 | 418.0 | 632.0 | 892.0 | 76.0 | 83.7 | 91.2 | 55.6 | 60.9 |
| V. varius | arboreal | 4820.0 | 261.0 | 357.0 | 618.0 | 752.0 | 56.7 | 67.6 | 95.4 | 55.5 | 55.9 |
| V. komodoensis | terrestrial | 30000.0 | unknown | unknown | 1011.0 | 1197.0 | 124.0 | 169.0 | 194.0 | 156.0 | 131.0 |
| V. komodoensis | terrestrial | 40000.0 | 402.0 | 790.0 | 1250.0 | 1460.0 | 182.0 | 249.0 | 189.0 | 188.0 | 189.0 |
| V. komodoensis | terrestrial | 40000.0 | 365.0 | 830.0 | 1195.0 | 1210.0 | 175.0 | 201.0 | 162.0 | 203.0 | 166.0 |
