# Supplementary material for: How to build your dragon: scaling of muscle architecture from the world’s smallest to the world’s largest monitor lizard
Source: Front Zool. 2016 Feb 18;13:8. doi: 10.1186/s12983-016-0141-5 (PMC4758084; doi:10.1186/s12983-016-0141-5)
Supplement: Additional file 3: Figure S1. — Different patterns of variation in aerobic and anaerobic muscle fibres within a single muscle belly for the iliofibularis (a-b), pubotibialis (c-d) and flexor tibialis internus (superficial) (e). Although not explicitly tested, the different bands of red and white muscle fibres suggest a regional arrangement of slow and fast muscle fibres, while the functional reason for this remains unclear. (PDF 1276 kb) [file 12983_2016_141_MOESM3_ESM.pdf]

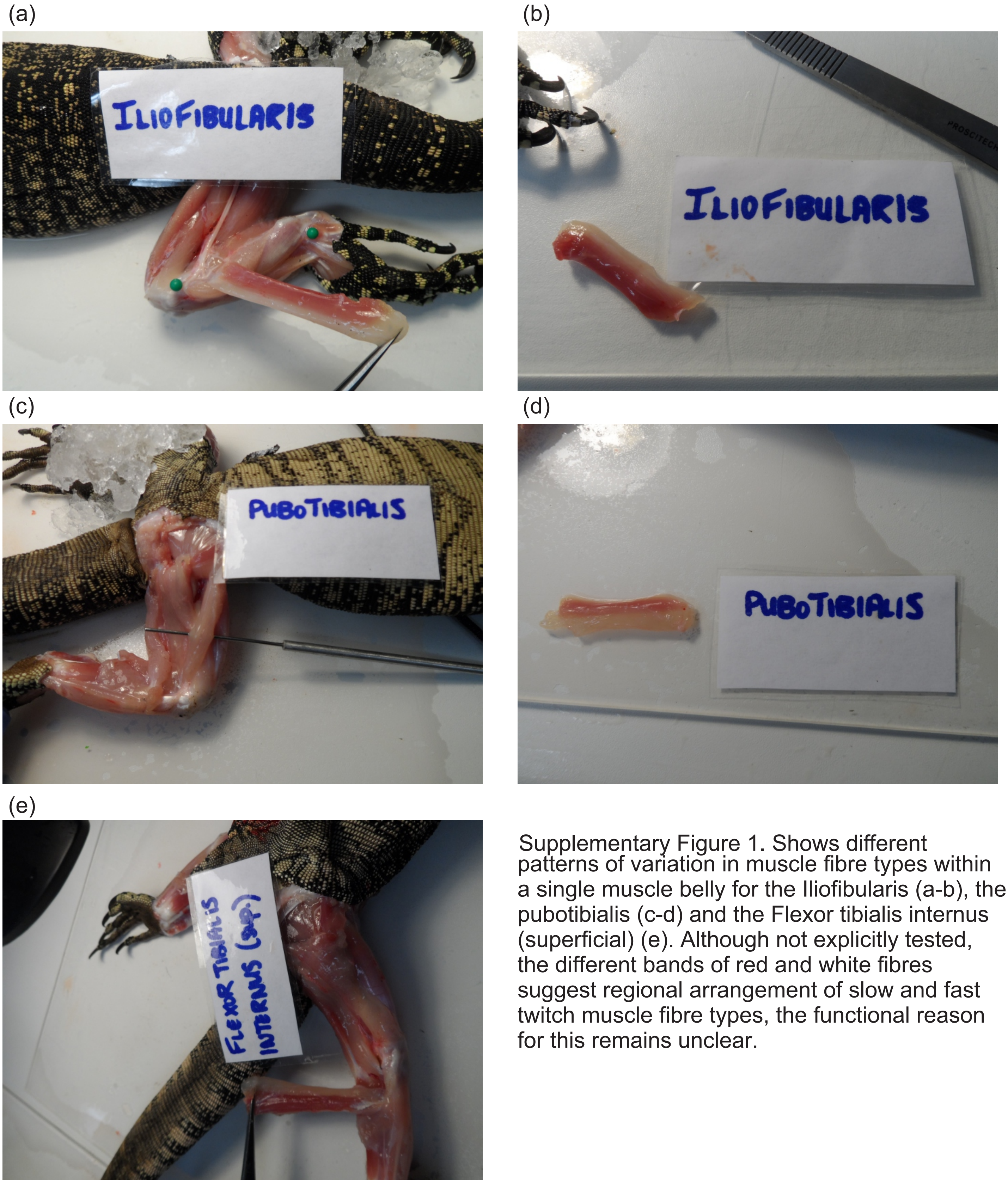

Supplementary Figure 1. Shows different patterns of variation in muscle fibre types within a single muscle belly for the Iliofibularis (a-b), the pubotibialis (c-d) and the Flexor tibialis internus (superficial) (e). Although not explicitly tested, the different bands of red and white fibres suggest regional arrangement of slow and fast twitch muscle fibre types, the functional reason for this remains unclear.
