# Supplementary material for: How to build your dragon: scaling of muscle architecture from the world’s smallest to the world’s largest monitor lizard
Source: Front Zool. 2016 Feb 18;13:8. doi: 10.1186/s12983-016-0141-5 (PMC4758084; doi:10.1186/s12983-016-0141-5)
Supplement: Additional file 4: Table S3. — Average muscle properties in the hindlimb of the varanid lizards. Pennation is an angle measured in degrees. (PDF 308 kb) [file 12983_2016_141_MOESM4_ESM.pdf]

Muscle	Species	Function	Body mass (kg)	M_{musc}/M %	$L_f/M^{0.33}$ %	Pennation	$PCSA/M^{0.66}$ %	$L_{ten\,(prox)}/M_{body}^{0.33}$ %	$L_{ten\,(dist)}/M_{body}^{0.33}$ %
AFEM	V. gouldii	protraction	0.443	0.111	NA	NA	NA	NA	NA
	V. komodoensis		36.667	0.265	3.897	NA	0.0079	NA	NA
	V. panoptes		1.609	0.204	3.769	NA	0.0045	NA	NA
	V. scalaris		0.158	0.073	NA	NA	NA	NA	NA
	V. tristis		0.158	0.141	3.596	NA	0.003	NA	NA
	V. varius		2.623	0.216	3.300	NA	0.006	NA	NA
AMB (D)	V. gouldii	knee extension	0.443	0.307	NA	NA	NA	NA	1.359
	V. komodoensis		36.667	0.293	4.168	NA	0.004	0.719	0.923
	V. panoptes		1.609	0.322	3.537	NA	0.003	0.965	1.509
	V. scalaris		0.158	0.179	NA	NA	NA	NA	1.377
	V. tristis		0.158	0.294	3.636	NA	0.003	1.197	1.449
	V. varius		2.623	0.278	3.620	NA	0.003	0.916	1.077
AMB (V)	V. gouldii	knee extension	0.443	NA	NA	NA	NA	NA	1.375
	V. komodoensis		36.667	0.355	3.156	NA	0.011	0.722	1.689
	V. panoptes		1.609	0.232	3.803	NA	0.006	0.922	1.633
	V. scalaris		0.158	NA	NA	NA	NA	NA	NA
	V. tristis		0.158	0.162	3.842	NA	0.004	0.960	1.296
	V. varius		2.623	0.151	3.492	NA	0.004	0.986	1.069
CFEMB	V. komodoensis	femur retraction, femur long axis rotation	40.000	0.181	2.881	NA	0.006	NA	NA
	V. panoptes		2.437	0.247	2.221	NA	0.011	NA	NA
	V. tristis		0.265	0.147	2.080	NA	0.007	NA	NA
	V. varius		4.820	0.385	2.464	NA	0.015	NA	NA
CFEML	V. brevicauda	femur retraction, femur long axis rotation, knee flexion	0.009	1.001	NA	NA	NA	NA	NA
	V. caudolineatus		0.013	0.828	NA	NA	NA	NA	NA
	V. eremius		0.016	0.946	NA	NA	NA	2.676	NA
	V. gouldii		0.443	2.169	6.012	20.556	0.037	4.927	NA
	V. komodoensis		36.667	1.157	3.186	24.556	0.040	1.875	NA
	V. panoptes		1.381	1.718	4.473	20.889	0.048	3.497	NA
	V. scalaris		0.158	1.198	5.439	22.333	0.022	1.869	NA
	V. tristis		0.158	2.930	5.190	21.778	0.055	1.933	NA

	V. varius		2.623	1.177	3.713	21.833	0.033	1.936	NA
EDL	V. brevicauda	ankle dorsiflexion	0.009	0.021	NA	NA	NA	NA	NA
	V. caudolineatus		0.013	0.052	NA	NA	NA	NA	NA
	V. eremius		0.016	0.089	NA	NA	NA	NA	NA
	V. gouldii		0.443	0.133	4.486	0.667	0.003	0.880	1.006
	V. komodoensis		36.667	0.087	3.687	0.222	0.002	1.155	0.949
	V. panoptes		1.381	0.136	4.497	0.389	0.003	1.097	0.903
	V. scalaris		0.158	0.080	4.079	0.000	0.002	0.570	0.959
	V. tristis		0.158	0.144	4.583	0.111	0.003	1.381	0.942
	V. varius		2.623	0.110	4.023	0.250	0.003	0.846	0.667
FTIB	V. gouldii	knee extension	0.443	0.109	1.936	14.000	0.005	NA	1.159
	V. komodoensis		36.667	0.105	1.927	23.889	0.006	NA	1.411
	V. panoptes		1.609	0.124	2.297	16.667	0.006	NA	1.498
	V. scalaris		0.158	0.053	1.802	20.000	0.003	NA	0.823
	V. tristis		0.158	0.580	2.030	16.000	0.027	NA	1.125
	V. varius		2.623	0.113	1.739	24.167	0.007	NA	1.226
FDL	V. komodoensis	ankle plantarflexion	40.000	0.136	3.065	NA	0.004	NA	1.464
	V. panoptes		2.437	0.090	3.101	NA	0.003	0.533	1.646
	V. tristis		0.265	0.023	2.552	NA	0.001	1.263	1.389
	V. varius		4.820	0.112	3.595	NA	0.003	NA	1.005
FTE	V. gouldii	knee flexion	0.443	0.172	NA	NA	NA	NA	1.266
	V. komodoensis		36.667	0.260	4.423	NA	0.008	1.482	1.263
	V. panoptes		1.609	0.243	4.489	NA	0.005	1.374	1.553
	V. scalaris		0.158	0.281	NA	NA	NA	NA	1.516
	V. tristis		0.158	0.198	4.193	NA	0.002	1.436	0.394
	V. varius		2.623	0.249	4.181	NA	0.005	1.575	1.360
FTI (D)	V. gouldii	flexion	0.443	0.083	4.997	0.556	0.002	NA	0.849
	V. komodoensis		36.667	0.068	3.974	0.444	0.002	NA	1.064
	V. panoptes		1.609	0.108	5.241	0.444	0.002	0.779	0.971
	V. scalaris		0.158	0.101	4.074	0.333	0.002	NA	0.855
	V. tristis		0.158	0.105	4.331	0.111	0.002	0.298	0.569
	V. varius		2.623	0.137	5.007	0.250	0.003	NA	0.599
FTI (S)	V. gouldii	flexion	0.443	0.148	5.969	0.889	0.002	NA	0.975
	V. komodoensis		36.667	0.244	4.595	1.222	0.005	NA	0.765

	V. panoptes		1.609	0.251	5.429	0.944	0.005	NA	1.393
	V. scalaris		0.158	0.108	4.139	0.667	0.002	NA	0.840
	V. tristis		0.158	0.160	4.070	0.222	0.004	NA	0.923
	V. varius		2.623	0.335	4.870	0.417	0.006	NA	0.660
GAST	V. brevicauda	ankle plantarflexion	0.009	0.701	NA	NA	NA	NA	1.261
	V. caudolineatus		0.013	0.120	NA	NA	NA	1.264	1.769
	V. eremius		0.016	0.178	NA	NA	NA	1.025	1.503
	V. gouldii		0.443	0.291	2.941	20.667	0.010	NA	1.200
	V. komodoensis		36.667	0.257	1.832	25.667	0.017	1.404	1.731
	V. panoptes		1.381	0.285	3.431	23.278	0.012	0.783	1.377
	V. scalaris		0.158	0.142	2.000	20.333	0.007	NA	1.200
	V. tristis		0.158	0.305	3.008	22.333	0.010	1.404	1.731
	V. varius		2.623	0.284	3.000	26.000	0.011	0.783	1.377
ILFEM	V. gouldii	femur abduction	0.443	0.054	1.938	1.444	0.003	NA	NA
	V. komodoensis		36.667	0.101	2.433	2.333	0.004	NA	NA
	V. panoptes		1.609	0.067	2.427	0.833	0.003	NA	NA
	V. scalaris		0.158	0.026	1.835	2.000	0.001	NA	NA
	V. tristis		0.158	0.048	2.029	1.111	0.002	NA	NA
	V. varius		2.623	0.069	2.523	0.417	0.003	NA	NA
ILFIB	V. brevicauda	knee flexion	0.009	0.052	NA	NA	NA	NA	NA
	V. caudolineatus		0.013	0.150	NA	NA	NA	NA	NA
	V. eremius		0.016	0.075	NA	NA	NA	NA	NA
	V. gouldii		0.443	0.166	4.225	1.667	0.004	NA	0.858
	V. komodoensis		36.667	0.234	3.988	0.222	0.006	NA	1.115
	V. panoptes		1.381	0.429	4.580	0.611	0.004	NA	1.339
	V. scalaris		0.158	0.112	4.860	1.333	0.002	NA	0.882
	V. tristis		0.158	0.182	4.654	0.444	0.004	NA	1.284
	V. varius		2.623	0.158	4.157	0.333	0.004	NA	1.070
ILTIB	V. brevicauda	knee extension	0.009	0.045	NA	NA	NA	NA	NA
	V. caudolineatus		0.013	0.064	NA	NA	NA	NA	NA
	V. eremius		0.016	0.102	NA	NA	NA	1.544	NA
	V. gouldii		0.443	0.172	3.384	2.833	0.005	1.398	1.320
	V. komodoensis		36.667	0.132	3.460	1.667	0.004	1.399	1.336
	V. panoptes		1.381	0.165	3.889	2.056	0.004	1.352	1.337

	V. scalaris		0.158	0.084	3.785	1.667	0.002	1.333	1.255
	V. tristis		0.158	0.128	3.888	0.889	0.003	1.219	1.288
	V. varius		2.623	0.128	3.966	1.500	0.003	1.472	1.227
PBREV	V. komodoensis	ankle dorsiflexion	40.000	0.091	2.149	24.000	0.005	0.866	2.203
	V. panoptes		2.437	0.072	3.294	22.000	0.002	0.970	0.673
	V. tristis		0.265	0.087	2.995	10.667	0.003	1.201	0.674
	V. varius		4.820	0.093	3.060	19.667	0.003	0.696	1.090
PLONG	V. gouldii	ankle plantarflexion	0.443	0.111	2.659	11.667	0.004	0.937	1.673
	V. komodoensis		36.667	0.087	2.308	17.556	0.004	0.904	1.203
	V. panoptes		1.381	0.142	3.020	15.333	0.005	1.071	1.849
	V. scalaris		0.158	0.076	2.449	12.333	0.003	1.110	1.518
	V. tristis		0.158	0.122	2.956	13.556	0.004	0.987	1.819
	V. varius		2.623	0.102	2.277	14.917	0.004	0.972	1.677
PIT	V. brevicauda	flexion	0.009	0.111	NA	NA	NA	NA	NA
	V. caudolineatus		0.013	0.228	NA	NA	NA	NA	NA
	V. eremius		0.016	0.325	NA	NA	NA	NA	NA
	V. gouldii		0.443	0.263	NA	NA	NA	NA	NA
	V. komodoensis		36.667	0.541	6.878	0.000	0.010	NA	0.680
	V. panoptes		1.381	0.378	5.939	NA	0.005	NA	0.618
	V. scalaris		0.158	0.249	NA	NA	NA	NA	NA
	V. tristis		0.158	0.457	5.729	NA	0.006	NA	0.718
	V. varius		2.623	0.471	5.429	NA	0.007	NA	0.588
PIF	V. gouldii	femur protraction	0.435	0.449	3.516	4.667	0.012	NA	NA
	V. komodoensis		36.667	0.487	3.212	2.444	0.011	NA	NA
	V. panoptes		1.609	0.391	3.526	2.278	0.012	NA	NA
	V. scalaris		0.158	0.331	2.703	2.667	0.006	NA	NA
	V. tristis		0.158	0.522	2.945	2.889	0.017	NA	NA
	V. varius		2.623	0.314	2.978	0.833	0.010	NA	NA
PTIB	V. gouldii	flexion	0.443	0.118	5.207	2.000	0.002	0.699	NA
	V. komodoensis		36.667	0.207	5.287	0.111	0.004	0.306	0.670
	V. panoptes		1.609	0.149	5.210	1.639	0.003	0.847	0.827
	V. scalaris		0.158	0.109	4.433	2.333	0.002	0.932	NA
	V. tristis		0.158	0.118	5.092	0.333	0.002	0.716	0.518
	V. varius		2.623	0.236	4.808	0.208	0.005	0.823	0.608

TIBA	V. komodoensis	ankle dorsiflexion	40.000	0.058	3.418	16.000	0.002	0.691	0.582
	V. panoptes		2.437	0.059	3.500	4.333	0.002	1.188	0.596
	V. tristis		0.265	0.170	3.986	16.333	0.004	0.657	1.226
	V. varius		4.820	0.071	3.406	9.667	0.002	0.869	0.871
