# Supplementary material for: How to build your dragon: scaling of muscle architecture from the world’s smallest to the world’s largest monitor lizard
Source: Front Zool. 2016 Feb 18;13:8. doi: 10.1186/s12983-016-0141-5 (PMC4758084; doi:10.1186/s12983-016-0141-5)
Supplement: Additional file 5: Table S4. — Results of RMA linear regression properties with body mass in the hindlimb of varanid lizards. Data presented is from a phylogenetically informed analysis on species means. (PDF 296 kb) [file 12983_2016_141_MOESM5_ESM.pdf]

A. Muscle mass vs Body Mass

	AFEM	AMB (D)	AMB (V)	CFEMB	CFEML	EDL	FTIB	FDL	FTE	FTI (D)	FTI (S)	GAST	ILFEM	ILFIB	ILTIB	PBREV	PLONG	PIT	PIF	PTIB	TIBA
Slope	1.206	1.053	1.061	1.072	1.043	1.134	0.993	1.369	1.065	0.961	1.139	1.021	1.198	1.127	1.103	1.010	1.006	1.126	1.015	1.107	0.802
Lower 95% CI	1.049	0.935	0.903	0.625	0.890	0.944	0.647	0.841	0.933	0.837	0.963	0.814	1.040	1.002	0.984	0.890	0.840	1.019	0.899	0.993	0.516
Upper 95% CI	1.386	1.186	1.248	1.839	1.223	1.363	1.523	2.229	1.214	1.102	1.347	1.282	1.380	1.269	1.236	1.146	1.205	1.244	1.144	1.234	1.247
R^2	0.990	0.993	0.997	0.965	0.968	0.957	0.899	0.972	0.991	0.990	0.985	0.934	0.990	0.982	0.984	0.998	0.983	0.987	0.992	0.994	0.978
P-value	0.015	0.287	0.205	0.631	0.550	0.153	0.966	0.068	0.249	0.459	0.088	0.834	0.019	0.050	0.085	0.763	0.930	0.028	0.754	0.051	0.119

B. Fascicle length vs Body Mass

	AFEM	AMB (D)	AMB (V)	CFEMB	CFEML	EDL	FTIB	FDL	FTE	FTI (D)	FTI (S)	GAST	ILFEM	ILFIB	ILTIB	PBREV	PLONG	PIT	PIF	PTIB	TIBA
Slope	0.319	0.328	0.269	0.399	0.236	0.310	0.351	0.377	0.315	0.315	0.339	0.304	0.384	0.301	0.320	0.271	0.317	0.338	0.350	0.344	0.303
Lower 95% CI	0.303	0.269	0.208	0.297	0.156	0.265	0.284	0.245	0.266	0.260	0.255	0.180	0.351	0.261	0.288	0.148	0.240	0.310	0.288	0.295	0.218
Upper 95% CI	0.335	0.400	0.348	0.537	0.357	0.364	0.433	0.579	0.374	0.383	0.450	0.513	0.420	0.347	0.355	0.497	0.419	0.367	0.425	0.402	0.423
R^2	1.000	0.996	0.993	0.990	0.906	0.987	0.976	0.979	0.997	0.980	0.957	0.844	0.996	0.989	0.994	0.955	0.959	0.999	0.980	0.988	0.988
P-value	0.050	0.911	0.037	0.064	0.084	0.336	0.466	0.282	0.326	0.552	0.806	0.694	0.006	0.133	0.456	0.272	0.709	0.326	0.453	0.482	0.355

C. Pennation vs Body Mass

	CFEML	EDL	FTIB	FTI (D)	FTI (S)	GAST	ILFEM	ILFIB	ILTIB	PBREV	PLONG	PIF	PTIB	TIBA
Slope	0.033	1.230	0.105	0.306	0.352	0.050	0.304	-0.403	0.199	0.174	0.073	-0.275	-0.623	0.308
Lower 95% CI	0.014	0.409	0.040	0.105	0.133	0.024	0.098	-0.167	0.064	0.053	0.038	-0.093	-0.236	0.050
Upper 95% CI	0.076	3.698	0.278	0.896	0.932	0.105	0.941	-0.975	0.617	0.571	0.140	-0.811	-1.644	1.917
R^2	0.539	0.073	0.337	0.134	0.332	0.671	0.002	0.478	0.003	0.761	0.742	0.114	0.339	0.010
P-value	0.001	0.035	0.035	0.879	0.880	0.001	0.875	1.000	0.352	0.149	0.002	1.000	1.000	0.928

D. PCSA vs Body Mass

	AFEM	AMB (D)	AMB (V)	CFEMB	CFEML	EDL	FTIB	FDL	FTE	FTI (D)	FTI (S)	GAST	ILFEM	ILFIB	ILTIB	PBREV	PLONG	PIT	PIF	PTIB	TIBA
Slope	0.801	0.709	0.797	0.689	0.699	0.681	0.687	0.999	0.896	0.631	0.795	0.812	0.813	0.803	0.734	0.781	0.703	0.709	0.689	0.771	0.519
Lower 95% CI	0.544	0.603	0.640	0.304	0.511	0.540	0.382	0.603	0.600	0.537	0.635	0.702	0.684	0.666	0.572	0.522	0.586	0.521	0.509	0.640	0.260
Upper 95% CI	1.179	0.834	0.992	1.561	0.957	0.857	1.237	1.656	1.339	0.742	0.997	0.939	0.967	0.968	0.941	1.167	0.843	0.966	0.933	0.928	1.036
R^2	0.983	0.997	0.995	0.910	0.947	0.972	0.799	0.970	0.982	0.986	0.973	0.989	0.984	0.982	0.967	0.982	0.983	0.989	0.951	0.982	0.940
P-value	0.118	0.144	0.029	0.850	0.640	0.731	0.865	0.036	0.043	0.479	0.075	0.013	0.023	0.037	0.298	0.170	0.387	0.387	0.714	0.074	0.251

E. Distal Moment Arm vs Body Mass

	AMB (D)	AMB (V)	CFEMB	EDL	FTIB	FDL	FTE	FTI (D)	FTI (S)	GAST	ILFIB	ILTIB	PBREV	PLONG	PIT	PTIB	TIBA
Slope	0.400	0.296	0.427	0.358	0.350	0.449	0.404	0.408	0.364	0.341	0.413	0.332	0.399	0.298	0.346	0.363	0.229
Lower 95% CI	0.241	0.157	0.081	0.270	0.233	0.093	0.221	0.295	0.219	0.250	0.283	0.263	0.216	0.196	0.088	0.250	0.044
Upper 95% CI	0.664	0.558	2.243	0.474	0.525	2.155	0.738	0.565	0.604	0.464	0.603	0.418	0.736	0.453	1.361	0.529	1.201
R^2	0.970	0.951	0.308	0.958	0.910	0.429	0.956	0.943	0.854	0.949	0.922	0.972	0.954	0.904	0.634	0.924	0.308
P-value	0.206	0.536	0.685	0.473	0.713	0.599	0.256	0.139	0.635	0.787	0.171	0.951	0.293	0.545	0.919	0.517	0.569

F. Proximal Moment Arm vs Body Mass

	EDL	FDL	GAST	PBREV	PLONG	TIBA
Slope	0.369	0.416	0.341	0.269	0.315	-0.140
Lower 95% CI	0.316	0.168	0.182	0.074	0.185	-0.007
Upper 95% CI	0.432	1.029	0.639	0.971	0.535	-2.718
R^2	0.987	0.885	0.766	0.699	0.840	0.418
P-value	0.107	0.398	0.898	0.628	0.822	1.000
